# Detection of Human Papillomavirus Genotypes and Epstein-Barr Virus in Nasopharyngeal Carcinomas at the Korle-Bu Teaching Hospital, Ghana

**DOI:** 10.1155/2017/2721367

**Published:** 2017-03-21

**Authors:** Du-Bois Asante, Richard Harry Asmah, Andrew Anthony Adjei, Foster Kyei, David Larbi Simpong, Charles Addoquaye Brown, Richard Kwasi Gyasi

**Affiliations:** ^1^Department of Pathology, University of Ghana School of Biomedical and Allied Health Sciences, College of Health Sciences, P.O. Box KB 4236, Korle-Bu, Accra, Ghana; ^2^Biomedical and Forensic Sciences, School of Biological Sciences, College of Agriculture and Natural Sciences, University of Cape Coast, Cape Coast, Ghana; ^3^Medical Laboratory Sciences, University of Ghana School of Biomedical and Allied Health Sciences, College of Health Sciences, P.O. Box KB 143, Korle-Bu, Accra, Ghana; ^4^Molecular Biology and Biotechnology, School of Biological Sciences, College of Agriculture and Natural Sciences, University of Cape Coast, Cape Coast, Ghana

## Abstract

Nasopharyngeal carcinomas (NPC) are endemic in Far East Asia and commonly harbour Epstein-Barr virus (EBV) which is known to serve as a key oncogenic promoter. Human papillomavirus (HPV) is known to contribute to the pathogenesis of NPC. However, in Ghana these two viruses have not been linked to NPC prevalence. This study was designed to determine the HPV genotypes and EBV involved in NPC tissue biopsies. A retrospective study design involving 72 formalin-fixed paraffin-embedded tissue (FFPET) samples of NPC from 2006 to 2012 were retrieved from the Department of Pathology, University of Ghana School of Biomedical and Allied Health Sciences. Sections were taken for histological analysis and for DNA lysate preparation. The DNA lysates were subjected to polymerase chain reaction (PCR) analysis to determine the presence of HPV genotypes and EBV. HPV specific primers were used to type for fourteen HPV genotypes (HPV-16, 18, 6/11, 31, 33, 35, 44, 42, 43, 45, 56, 52, 58, and 59). Out of the 72 NPC biopsies analyzed by PCR, EBV DNA was present in 18 (25%) cases and HPV DNA in 14 (19.23%). High risk HPV (HR-HPV) genotypes 18 and 31 were associated with the NPC. There were 3 (4.2%) cases of coinfection by both viruses. The EBV DNA present in the undifferentiated variant of the NPC and the histopathology of the NPC in Ghana is similar to the type described in endemic areas.

## 1. Introduction

Nasopharyngeal carcinoma (NPC) is classified as a malignant neoplasm, arising from the mucosal epithelium of the nasopharynx [1]. NPC has been classified by the World Health Organization (WHO) into three types: keratinizing squamous cell carcinoma (WHO type I), nonkeratinizing squamous cell carcinoma (WHO type II), and undifferentiated carcinoma (WHO type III). Types II and III are clearly associated with Epstein-Barr virus (EBV), whereas viral association with the NPC type I is more controversial [2, 3].

NPC is a commonly occurring tumour in individuals in the southern regions of China [4], the Eskimos and the Greenlanders [5]. Intermediate incidence has been reported in the Mediterranean basin [6], especially among the Arabic populations of North Africa, such as Morocco [7–9] and in East Africa (Kenya) [10]. However, low incidence has been reported in West Africa [11].

Men are twice as likely to develop NPC as women, and the incidence rate generally increases from ages 20 to approximately 50 with signs and symptoms presenting themselves as painless, enlarged cervical lymph nodes, nasal obstruction, epistaxis, diminished hearing, tinnitus, recurrent otitis media, cranial nerve dysfunction, sore throat, and headache [6, 12]. Oncogenic viruses play important roles in carcinogenesis, and their study has contributed towards elucidation of cell growth and pathways of cancer [13]. EBV and human papillomavirus (HPV) are oncogenic viruses that are capable of transforming normal cells into cancerous forms [1]. Retrospective and prospective epidemiological studies conducted elsewhere [14, 15] point out clearly the association between EBV and NPC.

EBV has been implicated in the pathogenesis of several human tumours such as the African form of Burkitt lymphoma, B-cell lymphomas in immunosuppressed individuals, nasopharyngeal and some gastric carcinomas, rare forms of T-cell lymphomas, and natural killer (NK) cell lymphomas [16]. Anim et al., in relating the high prevalence of Burkitt's lymphoma among Ghanaian children suggested that the much higher number of younger Ghanaian patients with NPC may be associated with early exposure to the Epstein-Barr virus which is known to be associated with both malignancies [6, 17].

HPV on the other hand has also been detected in a variety of head and neck tumours including NPC [18]. It is also strongly implicated in the pathogenesis of human cervical carcinoma [19]. They are classified according to their ability to transform epithelial cells. Low-risk HPV (LH-HPV) genotypes are associated with benign lesions such as Warts while infections with high risk HPV (HR-HPV) genotypes may progress to malignant lesions [7, 18].

Studies suggest that normal human pharyngeal epithelial cells, especially nasopharyngeal cells, could be susceptible to persistent HPV and EBV coinfections and that EBV and HR-HPV coinfections may play an important role in the initiation of neoplastic transformation of these cells [7]. Other studies have also provided evidence that viral infections by EBV and HPV are associated with NPC [7, 19–21]. In a study conducted in North Africa, NPC biopsies from patients harboured EBV and HR-HPV genotypes, suggesting the coexistence of EBV and HPV-HR types being an important factor in the initiation of NPC [7, 20].

In Ghana, studies on the prevalence of EBV and HPV genotypes and their involvement in NPC have not been carried out. Hence, this research work investigates the presence of these viral oncogenic DNAs associated with the NPC using molecular probes. Molecular analysis will help identify the viral genotypes responsible for the transformation of the nasopharyngeal epithelial cells to aid preventive and therapeutic strategies.

With the current prevalence rate of 29% of all head and neck cancers [22] and 1.2–1.3% of all cancers in Ghana [23], and the number of cases in Korle-Bu Teaching Hospital (KBTH), Ghana, increasing steadily over the last few years, a study in this regard is very necessary to provide baseline data on the presence of HPV and EBV DNA in NPC tissue samples.

## 2. Materials and Methods

### 2.1. Research Design

The study was a retrospective cross-sectional design with laboratory analysis to investigate the presence of the DNA of EBV and HPV genotypes from primary NPC archived tissue samples obtained from the Department of Pathology, University of Ghana, School of Biomedical and Allied Health Sciences (SBAHS), from January 2006 to December 2012. The inclusion criteria required a patient's age to be greater or equal to ten (≥10) and histopathologically diagnosed with primary tumour of NPC.

### 2.2. Sample Size and Study Site

A total of 72 formalin-fixed paraffin-embedded tissue (FFPET) samples from patients with histopathologically confirmed primary NPC at the Pathology Department, Korle-Bu Teaching Hospital (KBTH), Accra, Ghana, were used. The department serves as the main pathology unit of the hospital and receives referral cases from all over Ghana and other West African countries. It also serves as a teaching unit in pathology (histology, cytology, and immunology) for SBAHS and the School of Medicine and Dentistry. The patients in this study originated from various social and ethnic groups as well as geographically distinct areas from the Greater Accra region and the southern part of Ghana.

### 2.3. Data on Study Cases

The clinical information on gender, age, and diagnosis of the NPC cases was recorded.

### 2.4. Tissue Sectioning for Histological Analysis

After trimming of the 72 FFPET samples of NPC blocks obtained, 5 *μ*m sections of the tissue were taken using a microtome. The sections were then stained with haematoxylin and eosin (H&E), using an automated H&E staining system (Leica Auto Stainer XL). The H&E stained slides were reviewed under the light microscope at the Department of Pathology, SBAHS, to confirm the previous diagnosis. The histopathological types of NPCs were determined according to the WHO 1991 classification and consisted of type I (keratinizing squamous cell carcinoma), type II (differentiated nonkeratinizing carcinoma), and type III (undifferentiated nonkeratinizing carcinoma). The H&E stained tissue slides were also used primarily as microscopic controls to ensure that the tissue sections used in the PCR contained malignant tissue. Photomicrographs of the NPC subtypes were then captured using the digital binocular light microscope, OLYMPUS 500 microscope (Version E_LCmiro_09Okt, 2009).

### 2.5. Molecular Analysis

#### 2.5.1. Genomic DNA Extraction

Ten micrometre (10 *μ*m) sections of the tissue were taken using a microtome. To avoid carry-over of the samples and contamination, the microtome blade was changed after each section and all surfaces were also cleaned with xylene after each tissue section. The 10 *μ*m sections were placed into a sterile 2.0 mL microcentrifuge tube. The tissues were then digested in 250 *μ*L of digestion buffer by incubation for 16 hr at 55°C on a heat-block using the method described previously [24]. The digestion buffer contained 200 *μ*g/mL Proteinase K (Sigma, USA), 50 mM Tris-HCL (pH 8.5), 1 mM EDTA, and 0.5% Tween-20. After 16 hr of incubation, the proteinase K was inactivated at 100°C for 5 min on a heat-block. After cooling the samples to room temperature, they were centrifuged at 13000 rpm for 5 min in a microcentrifuge. The residues containing DNA lysate were pipetted out and the supernatant containing paraffin was discarded. The digested product of each sample was stored in a freezer at −20°C for future use. The residue obtained (DNA lysate) contained the DNA extract that was used as a source of DNA template for the PCR analysis.

#### 2.5.2. Human *β* Globulin PCR

The DNA extracts were analyzed for the presence of human genomic DNA by human *β*-globin DNA PCR using the primers PCO3+ and PCO4+. This PCR analysis served as quality control to ascertain the presence of human genomic DNA in the extract and also the purity of the DNA in the DNA tissue extract for PCR as described earlier [25].

#### 2.5.3. HPV Genotyping Using Nested Multiplex PCR

HPV genotypes in the NPC tissue biopsies were obtained by using a Nested Multiplex PCR as described previously [26]. In the first nested PCR, 4 *μ*L of DNA lysate obtained after the tissue digestion was used as a source of HPV DNA template, to amplify a 630 bp region in the E6/E7 region of the HPV genome using HPV general primers. For the second nested PCR, 2 *μ*L of the first nested product was used as the source of HPV DNA template to amplify specific region within the 630 bp E6/E7 using HPV genotype specific primers.

In the first nested PCR amplification reaction, a single consensus forward primer (GP-E6-3F) and two consensus reverse primers (GP-E7-5B and GP-E7-6B) were used. For a total volume of PCR reaction mix of 25 *μ*L, the reaction conditions were as follows: 3 *μ*L of 10x PCR buffer (Biopioneer Co., USA), 1.5 mM MgCl_2_ (Biopioneer Co., USA), 200 *μ*M of each of the four oligonucleotide triphosphates (dNTPs) (Sigma Co., USA), 3.75 pmol of each consensus primer and 0.625 units of Taq polymerase enzyme (Biopioneer Co., USA), and 1 *μ*L or 4 *μ*L of the DNA lysate, the tissue digested product (source of HPV DNA template). Nuclease-free water (Promega Co., USA) was used to make up the volume to 25 *μ*L. A master-mix was made without the DNA template, vortexed to mix, and then pulse centrifuged for 30 sec before being aliquoted out into the samples contained in the PCR tubes. The positive control was HPV-18 DNA-containing plasmid and negative control (25 *μ*L of primer mix). The controls were also taken through the same PCR reaction conditions as per samples. The PCRs were performed by the following cycling conditions for the first nested PCR: an initial denaturation at 94°C for 4 min, followed by 40 PCR cycles consisting of 1 min denaturation step at 94°C, annealing step at 40°C for 2 min and elongation step at 72°C for 2 min, and a single final elongation step at 72°C for 10 min. As an internal control for the first nested PCR, the positive and the negative control PCR products were analyzed on a 2% agarose gel for every batch of the first PCR. The presence of a 630 bp band of the positive control gel lane and a DNA absence in the negative gel lane signifies that that batch of PCR is fit to be continued for the second nested PCR.

For the second nested PCR, a total volume of PCR reaction mix of 25 *μ*L, the reaction conditions were as follows: 2 *μ*L from PCR products of the first nested (source of HPV DNA template) and 3.75 pmol of each of the genotype specific primers (forward and reverse). The HPV genotype specific primer sets of the following HPV genotypes; 6/11, 16, 18, 31, 33, 35, 44, 42, 43, 45, 56, 52, and 58, were used in three cocktails. Nuclease-free water was used to make up the volume to 25 *μ*L. The parameters for the first nested PCR reaction was maintained for the second nested PCR. 10 *μ*L of the PCR products was then separated on 2% agarose gel (Biopioneer Co., USA) thereafter in 1x TAE running buffer (Biopioneer Co., USA) by electrophoresis at 80 volts (Labnet International, Power station 300) using 2 *μ*L of blue/orange DNA loading dye (6x) (Promega Co., USA) and stained with 0.5 *μ*g/mL ethidium bromide (Life Technologies Co., USA). A hundred bp nucleotide sequence marker (Sigma Mo, USA) was run alongside the PCR products on the gel.

#### 2.5.4. EBV PCR Analysis

The EBV DNA sequence was accessed using NC_007605. Amplified product size of 229 bp for EBV was used for identification of EBV DNA using agarose gel electrophoresis. The same reaction mixture was carried out using a modified one-step PCR. The PCR cycling conditions for the one-step reaction are as follows: an initial denaturation and polymerase activation at 95°C for 10 min, followed by 35 PCR cycles consisting of a denaturation step at 95°C for 30 sec, an annealing step at 60°C for 30 sec, an elongation step at 72°C for 30 sec, and then a final extension step at 72°C for 5 min, as described previously [27].

#### 2.5.5. Analysis of PCR Amplicons

The amplification products (for both HPV and EBV) were analyzed by gel electrophoresis on 2% agarose gel and stained with 0.5 *μ*g/mL ethidium bromide. 10 *μ*L of each sample was added to 2 *μ*L of orange G (5x) gel loading dye for the electrophoresis. 100 bp DNA molecular weight marker (Sigma MO, USA) was run alongside the PCR products. The gel was prepared and electrophoresed in 1x TAE buffer using a minigel system at 120 V for 40 min and was optically visualized and photographed over a UV transilluminator (UVIsave gel documentation system, model GAS9200/1/2/3, Version 12), followed by image analysis.

#### 2.5.6. Ethical Issues

The study was approved by the College of Health Sciences Ethical and Protocol Review Committee, University of Ghana (ethical clearance protocol identification number: MSEt/M.7-P 4.6/2012-13).

#### 2.5.7. Statistical Analysis

The data obtained from the tests were analyzed using SPSS version 16 (Chicago, IL). Qualitative variables were summarized by proportions and percentages while means, medians, and standard deviations were used to summarize quantitative variables. A test of association between EBV and HPV infection and subject's clinical and demographic data were summarized by Fishers' exact test with corresponding 95% confident interval (CI) and Fisher' exact test's probability value (*P* value) was used to measure the statistical significance of the calculated odds ratio. The *P* value obtained as is deemed statistically significant when value of the corresponding association is less than or equal to 0.05.

## 3. Results

The demographic characteristics of the 72 NPC cases showed mean age of patients to be 36.33 ± 20.82 years (median age, 31.5 years; range 10–79 years), comprising 38 males and 34 females given a 1.12 : 1 male to female ratio.

### 3.1. Demographical Parameters of NPC Cases and EBV/HPV Presence

Table 1 shows the odds ratio (OR) for HPV and EBV DNA detection in the NPC cases, with corresponding 95% confidence interval (95% CI) according to their demographic parameters among 72 patients. Patients younger than or equal to 40 years (≤40 years), who accounted for 56.9% of our study subjects, were more likely to have EBV positive NPC tumours (OR = 0.58; 95% CI = 0.19–1.77), as compared to patients older than 40 years (>40 years). In contrast, patients > 40 years had higher HPV positive tumours (OR = 1.42; 95% CI = 0.44–4.57), as compared to patients ≤ 40 years. For gender, females accounted for 15.3% EBV positive NPC tumour cases and a corresponding 2.12-fold (95% CI = 0.71–6.30) higher EBV DNA prevalence as compared to the males (9.7%). Thus, women were more likely to have EBV positive NPCs than men. In contrast once more, males had a higher HPV positive frequency (13%) compared to females (5.6%) and a higher proportion [10 (71.4%) out of 14] of HPV DNAs realized, compared to women [4 (28.6%) out of 14]; thus male subjects were at an increased risk of HPV infection as compared to women, even though the differences in prevalence estimates were not statistically significant.

### 3.2. EBV/HPV DNA in NPC Cases

All NPC were regrouped into three histopathological types according to the WHO 1991 classification. This classification consisted of type I (keratinizing squamous cell carcinoma), type II (differentiated nonkeratinizing carcinoma), and type III (undifferentiated nonkeratinizing carcinoma) (Figure 3). WHO type III was the most frequent histological variant accounting for 56 (77.8%) NPC cases (Table 2). Positive total viral (HPV and EBV) DNA was detected in 29 (40.3%) samples, including cases of coinfection. Combined EBV and HPV infection was detected in 3 (4.2%) of the 72 cases, and of these, all 3 cases were of the undifferentiated variant of the NPC.

### 3.3. Association of NPC Histological Subtypes and EBV/HPV Presence

**Figure 1:**
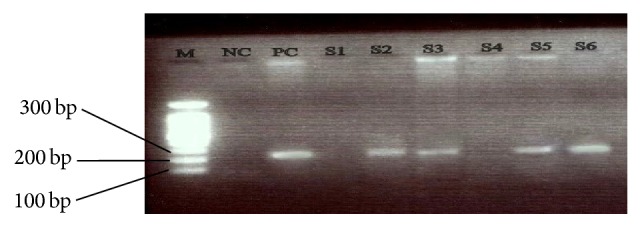
An electropherogram of Epstein-Barr virus PCR amplicons. The 229 bp fragment corresponds to the amplified EBV DNA. Lane M: 100 bp molecular size marker; NC: negative control (no DNA); PC: positive control; S2, S3, S5, and S6: EBV DNA positive samples; S1 and S4: EBV DNA negative samples.

The distribution of EBV DNA in the 18 EBV positive NPC cases (Figure 1) showed that the EBV DNA was only common in the NPC type III and was present in 25% of the entire NPC cases (18/72) and 32.14% (18/56) of the total number of type III cases obtained. None was detected in both types I and II. Out of the 14 HPV DNA positive cases detected, 10 were of the undifferentiated (type III); 3 were of the differentiated nonkeratinized (type II) and only 1 was of the keratinized type (type I). Although molecular analysis revealed that all the EBV positive NPC biopsies were of the type III, HPV testing showed the presence of HPV DNA in all 3 histological subtypes (Table 3). EBV/HPV had a relative risk (RR) of 1.2; thus NPC subjects have 1.2 times high risk of being infected with EBV than HPV. However, there was no statistically significant association between histological subtypes of NPC and EBV infection (*P* = 0.178) and also between histological subtypes of NPC and HPV infection (*P* = 0.595).

### 3.4. Frequency of HPV Genotypes

**Figure 2:**
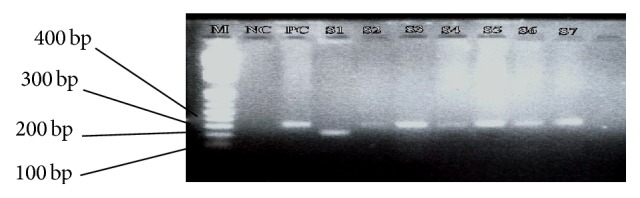
An electropherogram of 2nd nested PCR products of human papillomavirus. The 322 bp and 263 bp fragments corresponding to the amplified HPV-18 and 31 DNA, respectively. Lane M: 100 bp molecular size marker; NC: negative control (no DNA); PC: positive control (HPV-18 plasmid DNA); S1: HPV-31 DNA positive sample; S3, S4, S5, S6, and S7: HPV-18 DNA positive samples.

**Figure 3:**
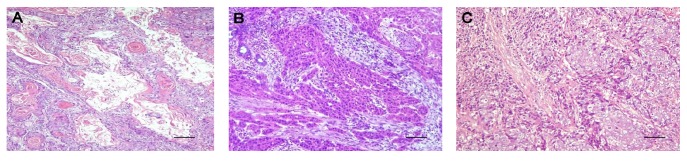
Photomicrograph of the NPC histological subtypes. (A) Type 1 NPC: differentiated malignant squamous cells showing keratin peals (keratin formation). (B) Type 2 NPC: the tumour cells are spindle shaped with elongated dark nuclei and tapering cytoplasm. (C) Showing a type 3 NPC. The tumour cells have vesicular nuclei and prominent nucleoli; the cytoplasm has indistinct cytoplasmic borders. The small basophilic cells depict lymphocytosis (H&E stain 100x). Bar = 20 *μ*m.

Out of the 14 HPV DNA positive cases, 13 (92.9%) were infected with HPV-18 genotype, and 1 (7.1%) was infected with HPV-31 (Figure 2). There were no HPV DNAs for the HPV genotypes 6/11, 16, 33, 35, 44, 42, 43, 45, 56, 52 58, and 59 in the study samples (Table 4).

## 4. Discussion

All the 72 archival tumour tissues were microscopically confirmed to contain malignant cells by H&E staining and were successfully regrouped under the WHO types I, II, and III of the NPC classification. From the human *β*-globulin PCR analysis, all the 72 NPC samples were positive for human *β*-globulin DNA.

Results showed a male preponderance with a male : female ratio of 1.12 : 1. This falls within the range of results obtained in Ghana [17, 28] and elsewhere [7, 21, 28–30] where the number of men was twice as or higher than that of women. The younger Ghanaian subjects (≤40 years), who accounted for 56.9% of our study subjects, were more likely to have EBV positive NPC tumours (OR = 0.58), as compared to patients older than 40 years (>40 years). This could be attributed to the fact that the virus is mostly acquired during the early stages of life and does not remain episomal, but rather infectious. In contrast, patients > 40 years had higher HPV positive tumours (OR = 1.42), as compared to patients ≤ 40 years. Thus, the virus could either be acquired during the later stage of life or it becomes infectious only during the later stages of life, mostly when the individual is immunosuppressed. Other research report showed equal distribution of NPC dominance in both younger (<40) and older (>40) subjects [7]. There is not enough information on age distribution and these oncogenic viruses in NPC research works.

For gender, females had higher (15.3%) EBV positive NPC tumour cases and a corresponding (OR = 2.12) than males (9.7%). Again, in contrast, males had a higher HPV positive frequency (13%) compared to females (5.6%). Thus, women were more likely to have EBV positive NPCs than men, with male subjects being at a higher risk of HPV infection as compared to women, even though the differences in prevalence estimates were not statistically significant. This disparity of viral tropism in gender group could be due to the route of entry and life style of the groups. Our result affirms other research works carried out elsewhere, where males had higher HPV positive cases (17), compared to females (7) [7]. This is also in consonance with results obtained from a recent work carried out using subset of head and neck squamous cell carcinomas (HNSCC) in Ghana, where out of 15 HPV positive cases, males had 14 compared to females (1) [31]. This sparks up the argument why young women and female adolescents are the only target group for vaccination against the oncogenic HR-HPV variants in some developing countries such as Ghana.

The observed results showed no significant differences between the oncogenic viral status with either age or gender in the NPC cases. These findings are in agreement with other studies carried out previously [7, 32, 33].

In endemic areas, the majority of the nasopharyngeal carcinomas are classified as nonkeratinizing WHO type II or undifferentiated type III tumours [34]. In contrast, in nonendemic countries in the Western hemisphere, it occurs sporadically and up to 50% of tumours are of the well differentiated squamous cell variant, WHO type I [35, 36].

Results from recent studies on NPC elsewhere pointed out that the nonkeratinizing type, particularly the undifferentiated carcinoma (WHO type III), was the most common histopathological type, followed by differentiated nonkeratinizing carcinoma (WHO type II) and then the keratinizing type (WHO type I) [28, 29], which agrees with findings from high incidence region of China where 84.6% of all histopathological types were nonkeratinizing and the keratinizing type accounted for only 5.8% [37]. In Africa, similar results were obtained in Morocco, where type III (88.9%) was the dominant histopathological variant, followed by type II (8.6%) and then type I (2.9%) [7].

In the present study, the observed results revealed that among the 72 cases, 56 (77.8%) were classified as type III, 13 (18.1%) as type II, and only 3 (4.1%) as type I.

The majority of our cases were of type III, followed by type II, whereas only three cases were found to be the type I variant. This suggest that the pathology of nasopharyngeal carcinomas in Ghana is indicative of the endemic type. This is in accordance with previous results from Anim et al. and Adam et al., who noted that the pathology of NPC in Ghana resembles that seen in endemic areas [17, 28].

The predominance of the undifferentiated type in most of the Ghanaian population demonstrates EBV genome (18/56) only in the type III NPC. Meanwhile, the relatively high prevalence of NPC cases in the younger Ghanaian subjects suggests that exposure to the EBV during the early ages of life could be a major contributing factor to NPC in Ghana. This confirms the earlier findings made by Anim et al., who stated that the close association between EBV and NPC on the one hand and Burkitt's lymphoma on the other could provide a common denominator in explaining the greater prevalence of NPC among young patients in Ghana [17].

The overall prevalence of EBV DNA in the studied NPC tissue samples was 25% (18 out of 72). Out of the 18 EBV DNA positive cases, results showed that the EBV DNA was only present in the NPC type III, thus 32.14% (18/56) of the total number of type III EBV positive cases obtained. None was seen in both types I and II. Although molecular analysis revealed that all the EBV positive NPC biopsies were of the type III, HPV testing revealed the presence of HPV DNA in all 3 histological subtypes. These figures are similar to those reported in Greece, where among 63 FFPET samples of NPC examined using PCR, EBV specific sequence was amplified in 20 (32%) and HPV in 12 (19%) of the samples [19]. This correlation between EBV and NPCs suggests that the virus plays a major role as an oncogenic driver in the pathogenesis of this tumour [7, 38]. Hundred percent correlation was obtained in other research works between EBV and the cancer [7, 39]. This was however not the case in the present study. This difference could be as a result of DNA degradation that occur in paraffin-embedded NPC biopsies as reported previously [39]. Thus, molecular results from these types of samples (FFPET) are critical as opposed to fresh samples.

The nonkeratinizing type (types II and III), particularly the undifferentiated histopathological type, has the strongest association with EBV infection [3, 28]. Results of our study showed that all the 18 EBV positive cases obtained were from the undifferentiated NPC type and thus affirm the fact that this NPC type is strongly associated with EBV. For distribution of HPV DNA, it was confirmed in at least one of each NPC histological type (10 type III, 3 type II, and 1 type I). Similar result was obtained in Morocco, where among 24 HPV positive cases, 22 were type III, 1 type II, and 1 type I [7]. In Iran, 4 out of 20 (20%) NPC cases contained HPV DNA sequences [21].

A total of fourteen (14) specific HPV genotype primers (HPV-6/11, 16, 18, 31, 33, 35, 44, 42, 43, 45, 56, 52, 58, and 59) were used in this study. This included both HR-HPV (16, 18, 31, 33, 35, 45, 52, 56, 58, and 59) and LR-HPV (6/11, 44, 42, and 43).

Out of the 14 HPV DNA positive cases, 13 (92.9%) were infected with HPV-18, and only 1 (7.1%) was infected with HPV-31; these are HR-HPV genotypes. This is in line with the research reported by Attoh et al. [40], where the prevalent HPV type in Ghanaian women with cervical cancer was HR-HPV type 18 (84%), but contrary to research report from Kaba et al., where HR-HPV type 16 was the prevalent HPV type (86.7%), using a subset of head and neck cancers from Ghanaian patients [31].

Although the study found only HPV-18 and HPV-31 in the NPCs, other research work reported on other HPV genotypes such as HPV-59, 16, 33, 35, 45, and HPV type 6/11 in NPC cases [7, 21]. The PCR method used in this study shows that oncogenic HPV DNA E6 and E7 from HPV-18 are in a significant proportion of the NPC cases studied. The detected genotypes in the study (HPV-18 and -31) are in the HR-HPV classification category which is reported risk factors associated with HNSCC, particularly in individuals without exposure to the widely known risk factors such as alcohol and/or tobacco [41]. It is also worth noting that HPV infection is a worldwide established aetiological agent of cervical cancers and the common HPV gene variants are HPV-16 and HPV-18 [42]. Our result had high number (13/14) of the common virulent HR-HPV gene variants in the study, supporting the fact that HR-HPV could be a cofactor in the pathogenesis of NPC.

In our study, EBV and HPV coinfection was detected in 3 (4.2%) of the 72 cases and was detected in the undifferentiated variant of the NPC. In USA, among 30 FFPET samples of NPC studied using the ligation-dependent polymerase chain reaction (LD-PCR), 2 samples were coinfected with HPV and EBV [43]. Also in Iran, among 20 NPC cases, 3 (15%) had coinfection with both viruses [21].

Research carried out in China revealed that, among 88 fresh NPC specimens from the Chinese population, coexistence of EBV and HPV DNA was observed in 42% of NPC samples [20], and similar results were also obtained in the Northern part of Africa, where coinfection by HPV and EBV was shown in 34% of the Moroccan patients [7]. This suggests that cumulatively the coexistence of EBV and HPV infection may be an important aetiological agent to consider in the pathogenesis of NPC, and although a couple of research works on HPV and EBV coinfection have been documented, the significance of the presence of both viruses in this tumours has not been elucidated.

In terms of histological types, EBV and HPV positive cases were seen mostly in the undifferentiated type of the NPCs (100% and 71.4%, resp.), followed by 21.4% and 7.5% for HPV only in type II and type I, respectively. However, there was no statistically significant association between histological subtypes of NPC and EBV infection and also between histological subtypes of NPC and HPV infection. Although some studies had no significant association to both EBV and HPV using FFPET samples [19], most studies using fresh NPC samples in Africa and elsewhere have shown a high level of association between NPC cases and EBV, but not HPV [7, 21, 28].

From the observed results, EBV/HPV had a relative risk of 1.2, which means that the NPC subjects have 1.2 times high risk of being infected with EBV than HPV. This fact is supported by the high number of EBV positive cases (25%) realized, as compared to HPV positive cases (19.4%) in the study.

Generally, we observed no statistically significant association between the oncogenic viral (EBV and HPV) infection and age, gender, or the tumour type. From the observed results, there appears to be a broad profile in the relationship between HPV, EBV, and NPC histological subtypes. In totality, this study would be the first to demonstrate EBV and HR-HPV (HPV-18 and HPV-31) genotypes in NPC cases in Ghana. Since no research data exists currently in the country on NPC and its association with the oncogenic viruses, it is hoped that the results of this study would act as the baseline for putting in place measures to monitor and control the disease.

## 5. Conclusion

Out of the 72 archival NPC biopsies screened, EBV DNA PCR amplification was achieved in 18 (25%) NPC cases and that of HPV DNA, in 14 (19.23%) cases. The HPV genotypes associated with the cancers were of the high risk types; HPV-18 and HPV-31. There appears to be a geographical difference between our results and those obtained from high risk areas. In conclusion, the histopathology of nasopharyngeal carcinoma in Ghana is of the type described in areas endemic for the disease, and they are associated with HPV and EBV in a minority of cases.

## Figures and Tables

**Table 1:** Relationship between demographical parameters of NPC cases and EBV/HPV presence *n* = 72.

Parameters	NPC cases *n* (%)	EBV+ *n* (%)	EBV− *n* (%)	OR (95% CI)	*P* value	HPV+ *n* (%)	HPV− *n* (%)	OR (95% CI)	*P* value
Age									
≤40 years	41 (56.9)	12 (16.7)	29 (40.3)	0.58	0.416	7 (9.7)	34 (47.2)	1.42	0.057
>40 years	31 (43.1)	6 (8.3)	25 (34.7)	(0.19–1.77)		7 (9.7)	24 (33.3)	(0.44–4.57)	
Gender									
Male	38 (52.8)	7 (9.7)	31 (43.1)	2.12	0.801	10 (13.9)	28 (38.9)	0.37	0.145
Female	34 (47.2)	11 (15.3)	23 (31.9)	(0.71–6.30)		4 (5.6)	30 (41.7)	(0.11–1.33)	

*Note*. HPV−: HPV negative NPC cases; HPV+: HPV positive NPC cases; EBV−: EBV negative NPC cases; EBV+: EBV positive NPC cases; *n*: number of cases; *N*: total number of cases; OR: odds ratio; CI: confidence interval.

**Table 2:** Summary of EBV/HPV DNA in NPC cases *n* = 72.

NPC subtype	NPC	HPV+/EBV+	HPV−/EBV−	Coinfection
	*n* (%)	*n* (%)	*n* (%)	*n* (%)
WHO type I	3 (4.2)	1 (1.4)	2 (2.8)	0 (0)
WHO type II	13 (18.0)	3 (4.2)	10 (13.9)	0 (0)
WHO type III	56 (77.8)	25 (34.7)	31 (43.0)	3 (4.2)
*Total*	*72 (100)*	*29 (40.3)*	*43 (59.7)*	*3 (4.2)*

Values are expressed as *n* (%).

**Table 3:** Association of NPC histological subtypes and EBV/HPV presence *n* = 72.

Oncogenic virus	WHO type I	WHO type II	WHO type III	Total	*P* value	RR
EBV	0	0	18	18 (25.0)	0.036	1.2
HPV	1	3	10	14 (19.4)	0.598	

*Note*. RR: relative risk.

**Table 4:** Frequency of HPV genotypes.

HPV genotype	Frequency (%)
HPV-18	13 (92.9)
HPV-31	1 (7.1)
*Total*	*14 (100.00)*
